# Effects of a Salutogenic Healthy Eating Program in Type 2 Diabetes (the SALUD Study): Protocol for a Randomized Controlled Trial

**DOI:** 10.2196/40490

**Published:** 2023-03-21

**Authors:** Kristel C M M Polhuis, Lenneke Vaandrager, Maria A Koelen, Johanna M Geleijnse, Sabita S Soedamah-Muthu

**Affiliations:** 1 Health & Society Wageningen University & Research Wageningen Netherlands; 2 Division of Human Nutrition and Health Wageningen University & Research Wageningen Netherlands; 3 Department of Medical and Clinical Psychology Center of Research on Psychological disorders and Somatic diseases (CoRPS) Tilburg University Tilburg Netherlands; 4 Institute for Food, Nutrition and Health (IFNH) University of Reading Reading United Kingdom

**Keywords:** salutogenesis, lifestyle, nutritional intervention, diabetes type 2, randomized controlled trial, RCT, patient centered, empowerment, resilience

## Abstract

**Background:**

Healthy eating can improve the course of type 2 diabetes mellitus (T2DM) considerably. As changing eating behaviors in everyday is challenging, there is a need for a nutritional strategy with an eye for everyday life of people with T2DM. A theory centered around the everyday life context is salutogenesis. Salutogenic principles have been operationalized in a new nutritional program for T2DM on food literacy and well-being: the Salutogenic Intervention for Type 2 Diabetes (SALUD) program.

**Objective:**

This study aims to describe the protocol of the invention study that will examine the quantitative and qualitative effects of the SALUD program.

**Methods:**

A semiblinded randomized controlled trial will be performed in the Netherlands. A sample size of 56 (including a 30% dropout rate) people with T2DM has been calculated, of whom half (n=28, 50%) will follow the SALUD program (intervention) and half (n=28, 50%) will receive usual care (control). Recruitment strategies consist of advertisement via local health care professionals, posters, social media, and local newspapers. The SALUD program consists of 12 weekly web-based group sessions under the supervision of a certified lifestyle coach. Fidelity of the delivery is guaranteed by selecting a salutogenic coach, use of an intervention manual, training of the coach, weekly evaluation forms, and recording several sessions. The theoretical salutogenic principle of the intervention is mobilizing 2 important psychosocial resources required for organizing healthy eating in everyday life: self-identity and social support. Measurements will be performed at 3 times: at baseline (T0), after 12 weeks (postintervention; T1), and after 24 weeks (follow-up; T2). The primary outcome is food literacy, measured with the self-perceived food literacy scale questionnaire (expected effect size=0.9). Secondary outcomes are self-efficacy, quality of life, sense of coherence, diet quality, body weight, BMI, and waist-hip ratio. All outcomes will be tested with linear mixed models, following an intention-to-treat approach and standard principles of randomized controlled trials. In addition, a qualitative analysis will be performed.

**Results:**

The proposed study will provide useful information on the effects of a salutogenic program on healthy eating and well-being in people with T2DM in everyday life. Recruitment started on October 1, 2021. The intervention participants followed the SALUD program between January and August, 2022. The acquisition of the data was completed on August 1, 2022; publications are expected in 2023.

**Conclusions:**

This study will be one of the first salutogenic interventions for T2DM, which will provide valuable information on what salutogenic intervention entail. The SALUD program may serve as a concrete, web-based tool. The combination of quantitative and qualitative measures allows a comprehensive evaluation of effects. These insights can be used for further optimalization of T2DM interventions.

**Trial Registration:**

Netherlands Trial Registry, NL8963; https://trialsearch.who.int/Trial2.aspx?TrialID=NL8963

**International Registered Report Identifier (IRRID):**

DERR1-10.2196/40490

## Introduction

### Background

Healthy eating is important for the disease course and quality of life (QoL) of people with type 2 diabetes mellitus (T2DM) [1]. Healthy eating may lower hemoglobin A_1c_ (HbA_1c_) to a similar extent as medical treatments for T2DM [2,3]. In some cases, healthy eating can put the disease in remission [4-12]. However, people with T2DM indicated that implementing healthy eating recommendations in their everyday lives is challenging [5,7,8].

Several strategies are available to support people with T2DM with healthy eating. The strategies approach healthy eating in dissimilar manners that could be broadly categorized as dietary prescription, lifestyle counseling, and behavioral therapies [13]. Dietary prescription is characterized by a “top-down” and biomedical approach: the therapist actively provides the solution in the form of a strict calorie-restricted diet, and the main goal is to achieve weight loss or improved HbA_1c_ levels (eg, [12]). In lifestyle counseling, the participant has a more active role: the therapist provides lifestyle education, meal plan templates, and personalized advice to implement nutritional recommendations. The main goal is adherence to a healthy lifestyle (eg, the Look AHEAD [Action for Health in Diabetes] trial [14] and the Diabetes Remission Clinical Trial [DIRECT]; [15]). Behavioral counseling is characterized by a more “bottom-up” and holistic approach: the starting point is the participant’s preferences; the goals and approaches are collaboratively planned. The therapist has an enabling role in the process: stimulating the participant’s problem-solving abilities and self-efficacy by motivating and supporting the participant. The main goal is achieving a mindset that favors healthy lifestyle behaviors (eg, [16]).

All of these strategies have been successful in initiating health benefits such as weight loss and improved HbA_1c_ levels [12,16,17] but have struggled with maintaining the benefits in the long term [8,18-21]. Even after the most well-known T2DM interventions—the Look AHEAD [14] and the DIRECT trial [15]—health benefits gradually disappeared. The Look AHEAD trial (restricted caloric intake using portion-controlled meal plans, calorie-counting techniques, and meal replacements combined with moderate physical activity) demonstrated average weight loss of 8.6% (SD 6.9%) in the intervention participants, but half of the initial weight loss was regained after 5 years of follow-up [22,23]. The DIRECT trial (a very low–calorie diet, followed by stepwise food reintroduction) demonstrated that almost half of intervention participants achieved T2DM remission at 12 months, but this percentage declined to 36% at 24 months [24].

Most previous studies used dietary description or lifestyle counseling strategies. These strategies either included strict (ie, very low–calorie) diets or were performed in highly controlled research settings (intensive intervention regarding diet and physical activity combined with frequent contact with health care providers) [12,25,26]. However, people may experience difficulties when they return from these clinical and controlled research settings to their everyday lives. In everyday life, healthy eating requires a scale of personal and social resources, ranging from practical cooking skills to personal agency [27-29]. It is not only about *what* people eat but also *why* and *how* they eat: the ability to prepare food, social economic resources, the food environments, social support, self-image, mental health, etc. This may explain why the Mediterranean diet is considered as one of the healthiest diets with (long-term) health benefits [30]; the Mediterranean diet is not a strict diet, rather *a way of life* that includes seasonal cooking, freshly cooked meals, and socializing with others [31].

### Objectives

Hence, there seems to be a need for a strategy with an eye for everyday life of people with T2DM to promote long-term healthy eating. A health theory centered around the everyday life context is salutogenesis. Applying salutogenesis to nutritional research leads to the assumption that healthy eating is not regarded as a central goal in life but as a resource for greater enjoyment of life [32]. It requires to accept a holistic viewpoint on health and real-life complexity as starting points for conducting and evaluating nutritional interventions. In salutogenesis, health results from the continuous everyday life interactions between the individual and the social, economic, cultural, physical, mental, and biochemical stressors [33]. The central concept in salutogenesis is the sense of coherence (SoC), which is the individual capability to mobilize and use health-promoting resources [33]. Understanding the *resources* that facilitate coping with these stressors in a health-promoting manner can help to unravel underlying principles of creating health [34]. Salutogenesis guided the development of the Salutogenic Intervention for Type 2 Diabetes (SALUD) program [35].

The aim of this study protocol is to outline the randomized controlled trial (RCT) in which the SALUD program is compared with the Dutch usual care for T2DM. The theoretical principle behind the program is mobilizing 2 important psychosocial resources required for organizing healthy eating and improved well-being in everyday life: self-identity and social support [35]. The present hypothesis is that mobilizing these resources will initiate a process of empowerment, leading to improved food literacy (direct effect) and improved health (indirect effects). Food literacy is the capability to navigate different food situations to make healthy eating choices [36], which is important for healthy eating and well-being in the long term. It is a measure that combines all relevant aspects for healthy eating in everyday life, that is, knowledge, skills, and behaviors. Hence, the primary objective is to determine the effect of the SALUD intervention on food literacy. Diet quality as well as several physical and psychosocial outcomes will be evaluated as secondary outcomes.

## Methods

### Study Design

**Figure 1:**
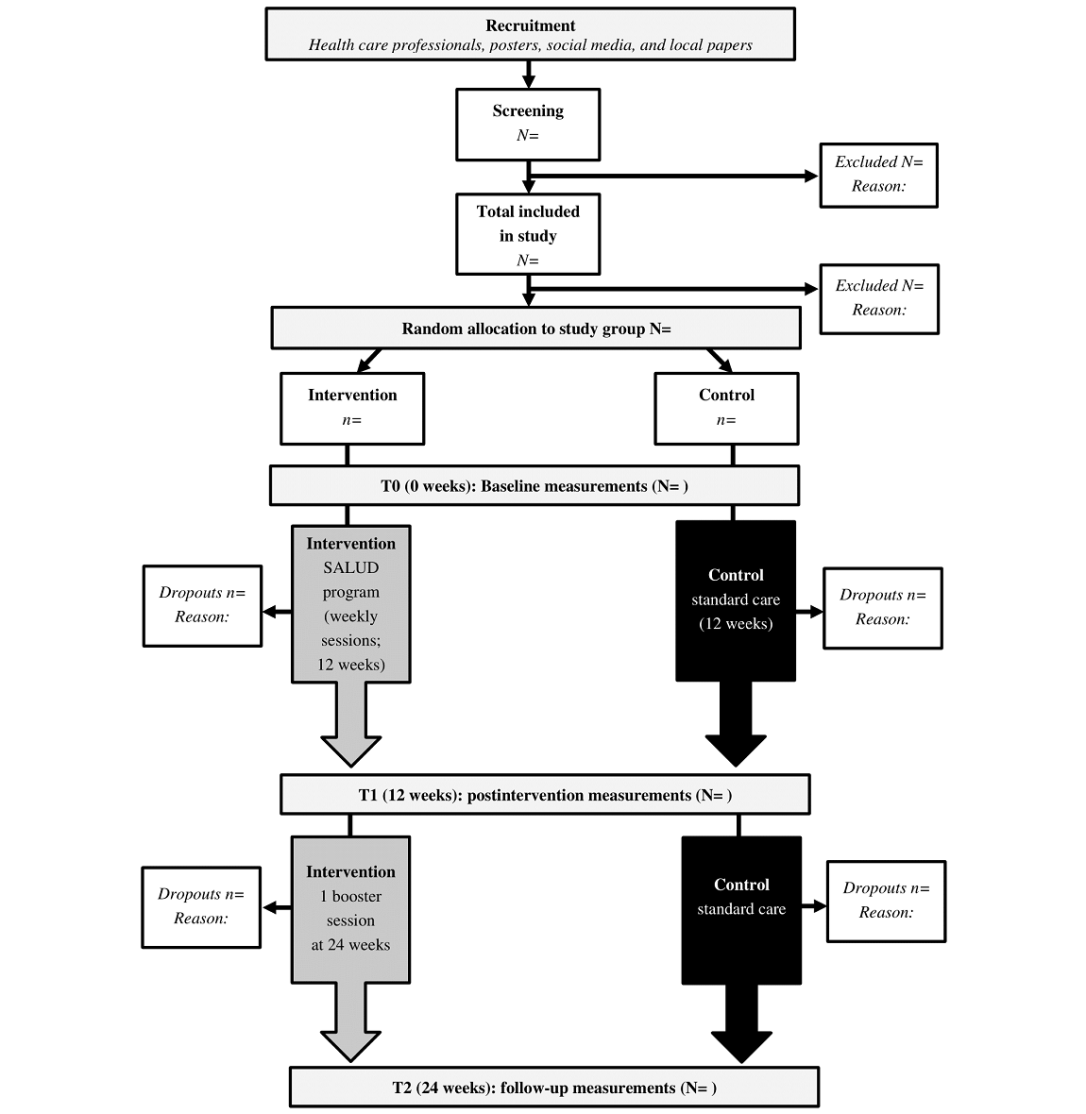
Salutogenic Intervention for Type 2 Diabetes (SALUD) study procedure.

A 2-arm, semiblinded RCT will be conducted with 56 people with T2DM in the Netherlands. The whole study duration will be 24 weeks, with measurements planned at baseline (T0), after 12 weeks (postintervention; T1), and 24 weeks (follow-up; T2). Participants will be randomly allocated to the intervention group (SALUD program) or to the control group (usual T2DM care [37]). The SALUD program will consist of 1 individual intake session (at the start), group sessions for 12 consecutive weeks, and 1 booster session (at 24 weeks). The study procedure is outlined in Figure 1.

### Ethics Approval

Medical Ethical Committee Oost-Nederland has granted medical ethics approval for this study on August 8, 2021 (Medical Ethical Committee number 2021-12949).

### Trial Registration

The study has been registered at Netherlands Trial Registry with registration number NL8963 [38] (registered on October 12, 2020).

### Participants

The target population consists of Dutch-speaking men and women with T2DM. Inclusion criteria are as follows: officially diagnosed with T2DM by a general practitioner (GP); aged ≥18-75 years; fluent in Dutch, competent to make own decisions; and in possession of a computer device with internet connection. Exclusion criteria are as follows: pregnant, lactating, or intention to become pregnant in the upcoming 6 months; severe chronic illnesses or condition or comorbidities (other than T2DM, eg, kidney failure, severe form of chronic obstructive pulmonary disease [gold III or IV], heart failure [class 2-4], cancer, dementia, or severe depression); bariatric surgery in the past; and eating disorders (eg, anorexia or bulimia).

### Recruitment

Participants are recruited via posters in GP practices, advertisements in local papers, social media, and websites of national diabetes organizations. In addition, several local GPs and practice nurses approach clients during regular health appointments or via phone or mail. Before commencement, all participants receive the information brochure that explains the study in detail and a phone call from the investigator for further explanation. At least 7 days after the phone call, participants sign an informed consent form and send it to the investigators before the start of the study. Participants are informed (written and verbally) that they will be randomly allocated to either the intervention or control group.

### Randomization and Blinding

Randomization of participants will be performed by an independent researcher (not involved in the study) with use of a web-based randomization tool. The data analysis for the primary outcome will be run blinded by an independent researcher. Treatment codes will be broken after finalization of the data analyses. Participants and researchers are not blinded during the measurements owing to the nature of the intervention.

### Intervention: SALUD Program

#### Theory Operationalization

For the development of the SALUD program, 3 main principles of salutogenesis were operationalized:

- The participant as a whole: in salutogenesis, health is a complex and dynamic concept incorporating multiple aspects of well-being that relate to the whole person [39]. This requires interventions that aim to improve multiple aspects of health. Hence, in the SALUD program, physical and psychosocial health are valued equally.
- The participant’s active involvement: to facilitate the mobilization of health resources, intervention strategies should be adjusted to real life to increase the chance of successful implementation of newly adopted behaviors in everyday life. This can only be done successfully and respectfully when people with T2DM and their health care providers are actively involved in the development of interventions. Hence, people with T2DM, health care professionals, and scientists of different disciplines provided their perspectives multiple times during intervention development (the participatory developmental process has been described by Polhuis et al [35]). The participatory developmental process revealed that self-identity, social support, food literacy, disease acceptance, and stress management were crucial resources to enable healthy eating in everyday life.
- The participant’s individual learning process: salutogenesis complements traditional information-providing approaches by supporting individuals in a learning process to mobilize personal and environmental health-promoting resources to cope with stressors. Hence, the strategy of the intervention was to enhance the generalized resistance resources (GRRs) and specific resistance resources (SRRs) for enabling healthy eating through a reflective and empowering approach. The main strategy was based on mobilizing the GRRs self-identity and social support; session themes were based on the identified SRRs food literacy, disease acceptance, and stress management (Table 1).

**Table 1:** Summary of the Salutogenic Intervention for Type 2 Diabetes (SALUD) program sessions.

Session topic	Setting	Goals
0. Intake	Coach and participant one on one (web-based)	Getting to know the participant; personal history, motivations, and goals
1. Building trust	Coach and peer-group (web-based)	Establishing a safe place Social bonding
2. Goal setting	Coach and peer-group (web-based)	Formulating at least one personal Specific, Measurable, Achievable, Relevant, and Time-Bound (SMART) health goal
3. Food literacy I	Coach and peer-group (web-based)	Reflection Question hour with a dietician Setting up an individualized diabetes-proof week menu Practical skills for meal planning, grocery shopping, and cooking
4. Food literacy II	Coach and peer-group (web-based)	Reflection Learning and sharing recipes (SALUD recipe book)
5. Stress management	Coach and peer-group (web-based)	Reflection Stress management Diabetes-related stress Mindfulness exercises (body scan and breathing exercises)
6. Nature break	Coach and peer-group (web-based)	Reflection Stress management Boosting positive emotions A mindful, individual walk outside (20 minutes)
7. Measuring progress	Coach and peer-group (web-based)	In-depth reflection, evaluation, and adjustment of health goals Social support
8. Social support	Coach and peer-group (web-based)	Involve partner or friend in the health process Opportunity for partners or friends to ask questions about program or health Social support
9. Self-identity	G Coach and peer-group (web-based)	Reflection Why do you eat what you eat? Values and life goals Disease acceptance Social support: question hour with role model
10. Open session	Coach and peer-group (web-based)	The participants choose the topic of this session during session 7
11. Measuring progress	Coach and peer-group (web-based)	In-depth reflection, evaluation, and adjustment of health goals Social support Long-term health strategies
12. Festive closure	Coach and peer-group (web-based)	Reflection on personal learning trajectory Boosting positive emotions Long-term health strategies
13. Booster session	Coach and peer-group (web-based)	Reflection and sharing of experiences over the past 12 weeks Social bonding Long-term commitment to a healthy lifestyle

#### Setting

The SALUD program takes place in a web-based group setting (7-8 participants per group) under supervision of a certified and experienced lifestyle coach. The communication platform Microsoft Teams (Microsoft) is used for hosting the sessions. The job of lifestyle coach in the Netherlands entails coaching and motivating people to sustainable lifestyle changes regarding nutrition, physical activity, sleep, and stress. Certified lifestyle coaches are extensively trained in various communication and coaching techniques. They have basic knowledge of lifestyle-related diseases, physiology, and nutrition. The current coach has been externally hired for the study by the authors and is not part of the research team. The coach is specifically experienced in group coaching (>5 years), particularly people with T2DM. The participants will be divided into separate groups on different days (based on their availability), all under supervision of the same coach.

#### Content

The SALUD program consists of 1 individual intake session (at the start), 12 weekly group sessions, and 1 booster session. Each weekly session has its own theme and goals (summary in Table 1). The sessions’ themes are based on the GRRs and SRRs that were identified during the participatory intervention development: self-identity, social support, food literacy, disease acceptance, and stress management. A “learning by experience” approach will be used to equip participants with practical tools and skills. The coach uses several reflective (on- and off-screen) exercises related to diet, health, stress, and life during each session. The sessions are intended to be highly interactive and informal. Most sessions end with a practical home exercise (eg, “cook a new recipe this week”). A registered dietician will be invited to 1 of the sessions to answer specific nutrition-related questions. In 2 of the sessions, participants are encouraged to bring an important person (their best friend, partner, neighbor, or family member). Session 10 is deliberately left “open” to provide the participants with the opportunity to choose a topic based on their needs. More details about the intervention content can be found in the study by Polhuis et al [35].

#### Fidelity of Delivery

Fidelity of the intervention delivery is assured by the following:

- selecting a coach in line with salutogenic principles, that is, whole person view on health and a participatory and empowering way of coaching
- using a program manual in which the program’s core (salutogenic) values, the sessions, the exercises, and goals are outlined
- thoroughly discussing with the coach about the (salutogenic) approach of the program before the start of the study
- weekly evaluating forms that will be filled in by the coach after each session to keep track of deviations from the manual and feedback of participants
- weekly checkups between the coach and the principal investigator (KP) to evaluate the previous session and to discuss the upcoming session
- recording a (small) number of sessions (with permission of the participants).

### Control: Usual T2DM Care

The participants in the control group receive the usual Dutch T2DM care according to the Dutch General Practitioners Association (Nederlands Huisartsen Genootschap) protocols [37]. The usual care of T2DM is based on medical treatment, education, and lifestyle advice. Newly diagnosed persons and persons who require insulin administration are referred to a dietician by the GP. In some cases, the GP recommends a referral to a psychologist for help with dietary advice or to a physical therapist to help with physical activity. The GP refers to a specialist in case of complications (such as retinopathy and nephropathy). Generally, a person with T2DM has 4 quarterly checkups with the practice nurse and 1 yearly checkup with the GP. However, the exact frequency of the appointments depends on the specific agreements between the health care provider and the patient.

### Quantitative Evaluation

**Figure 2:**
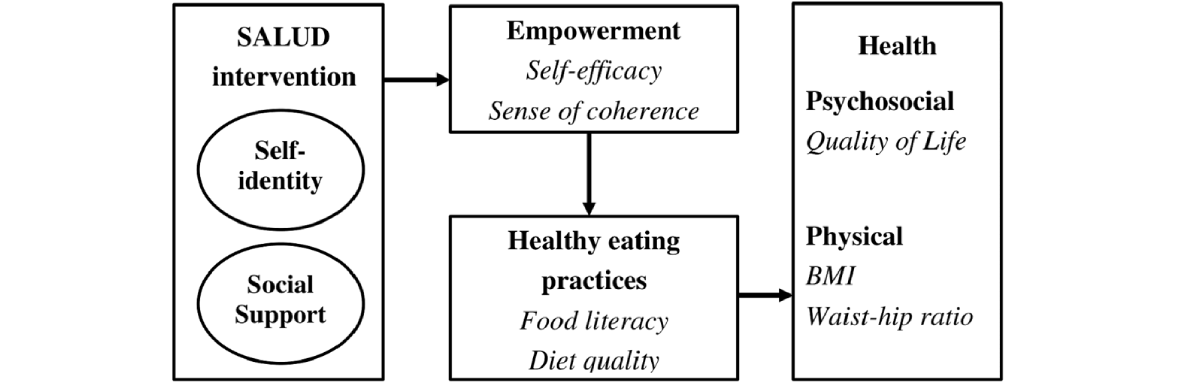
Proposed mechanism of action of the Salutogenic Intervention for Type 2 Diabetes (SALUD) program.

The primary outcome is food literacy; secondary outcomes are self-efficacy, QoL, SoC, diet quality (Dutch Healthy Diet Index 2015 [DHD-15 index]), body weight, BMI, and waist-hip ratio (WHR). Self-efficacy and SoC are measured to evaluate the empowerment process of the SALUD program; diet quality to assess changes in nutritional intake; QoL to assess effects on psychosocial health; and body weight, BMI, and WHR to assess the effects on physical health (Figure 2). All measurements take place at the participants’ homes.

#### Food Literacy (Self-perceived Food Literacy Scale)

The self-perceived food literacy scale (SPFLS) [36] is used to determine the capability to make healthy food choices in different contexts, settings, and situations. The SPFLS is a relatively new tool developed by Dutch researchers and validated among healthy Dutch adults (with a high internal reliability; Cronbach α=.80) [36] as well in people who underwent kidney transplant [36,40]. In the general population and in the patients who underwent kidney transplant, higher food literacy was associated with a healthier nutritional intake [36,40]. The SPFLS scale is proven to be capable of distinguishing between different populations [36,40]. The SPFLS contains 29 items related to 8 domains: food preparation skills, resilience and resistance, examining food labels, daily food planning, healthy budgeting, and healthy food stockpiling, healthy snack manners, and social and conscious eating. Response options include a 5-point Likert scale (1=“not at all/never” to 5=“yes/always”), eventually providing a score between 29 and 145. A higher score indicates higher food literacy. Domain scores and a total score are computed for each participant. Permission to use the SPFLS in this research has been formally granted by the developers of the SPFLS.

#### Self-efficacy (Dutch General Self-Efficacy Scale)

The Dutch General Self-Efficacy Scale (DGSES) [41] is a unidimensional free accessible questionnaire that measures the confidence someone has in their own abilities to cope with external challenges. The original DGSES was developed in German and is currently adapted to 32 languages [41]. Internal consistencies yielded typically Cronbach α between .76 and .90 [41]. Criterion validity is documented in numerous correlation [41]. The DGSES consists of 10 questions related to how people generally think and act. The response options include a 4-point Likert scale (1=completely false; 4=completely true), eventually providing a score between 10 and 40, with a higher score indicating higher self-efficacy. A total score will be computed for each participant.

#### QoL (World Health Organization Quality of Life Questionnaire)

The Dutch and abbreviated version of the World Health Organization Quality of Life-100 (WHOQOL-100) questionnaire is used to determine how people perceived their QoL over the last 2 weeks [42]. The abbreviated WHOQOL-100 is known as WHOQOL-BREF. The WHOQOL-BREF has been validated by comparing it with the original WHOQOL-100 domain scores (domains scores correlate at around 0.9 with the original) [43]. Internal validity is high (Cronbach α>.70) [44]. The WHOQOL-BREF contains a total of 26 questions that assess 5 domains of health and well-being: general perceived QoL, physical health, psychological health, social relationships, and environment. Response option includes a 5-point Likert scale (1=not at all; 5=extremely true), eventually providing a score between 26 and 130. Higher scores denote better QoL. A score is computed for each QoL domain as well as a total QoL score. Permission to use the Dutch version has been granted by the World Health Organization.

#### SoC (13 Items of the Orientation to Life Questionnaire)

The Dutch version of the 13 items of the Orientation to Life Questionnaire (SoC-13) is used to assess the participants’ SoC [34]. The SoC measures to what extent participants perceive the world as comprehensible, meaningful, and manageable. The SoC-13 has been validated in multiple countries with a high internal validity (Cronbach α between .70 and .92 [45]). The Dutch version is validated among Dutch adults (Cronbach α=.86; based on the validation study of Swan, E, unpublished data, June 2013). Response options include a 7-point Likert scale (1=very often; 7=almost never), eventually proving a score between 13 and 91, with a higher score indicating a stronger SoC. Domain scores for manageability, comprehensibility, and meaningfulness as well as a total SoC score are computed for each participant. Permission to use the SoC-13 has been granted to the authors.

#### Diet Quality (DHD-15 Index)

The DHD-15 index is used to assess the diet quality via a scoring system based on the Dutch food-based dietary guidelines [46]. The DHD-15 index is a relatively short food frequency questionnaire consisting of 40 questions. The DHD-15 index has been validated with 24-hour recalls and food frequency questionnaire data among Dutch adults with a Dutch dietary pattern [46]. The DHD-15 index calculates a score for 16 food components: vegetables, fruit, wholegrain products, legumes, nuts, dairy, fish, tea, fats and oils, coffee, red meat, processed meat, sweetened beverages and fruit juices, alcohol, salt, and unhealthy food products (snacks and cookies). Each component is scored on a scale ranging from 0 (implies nonadherence) to 10 (optimal adherence), eventually providing a total score between 0 and 160. A higher score indicates a more optimal diet quality (ie, a diet in line with the Dutch dietary guidelines). For the calculation of the scores, specific cutoff and threshold values were used as suggested by Looman et al [46]. A separate score for each component as well as a total score will be calculated. Formal permission to use the DHD-15 index was granted by the developers to the authors.

#### Anthropometry

Body weight is measured at the participant’s home with use of a scale (type: UC-411PBT-C) that measures to 2 decimals precisely. The scale will be calibrated before each measurement. The researcher makes sure the measurements are performed in similar circumstances (time of the day, placement of the scale, and layers of clothing). In addition to weight, height is measured once (during the first measurement). The scale calculates BMI (BMI [kg/m^2^]*=body weight [kg]/height [m]^2^*) and estimates fat percentage (%), muscle mass (kg), and basal metabolic rate (kcal).

#### Waist Circumference and WHR

Waist circumference and WHR are simple and useful measures of fat distribution. The measurements are performed according to the instructions of the World Health Organization [47]. In short, the participant must stand with arms at the sides, feet positioned close together, and weight evenly distributed across both feet. The measurement is performed at the end of a normal expiration. The researcher uses a measuring tape to measure the midpoint between the lower margin of the least palpable rib and the top of the iliac crest to determine waist circumference. Hip circumference is measured around the widest portion of the buttocks, with the tape parallel to the floor. All measurements are performed at the same time of the day (morning, afternoon, or evening). WHR is calculated using the following formula: *WHR=waist circumference (cm)/hip circumference (cm)*. A healthy WHR is considered ≤0.90 for men and ≤0.85 for women [47].

#### Participant Characteristics

A questionnaire consisting of personal contact details (name, age, address, phone number, and email), living situation, ethnicity, educational level, and clinical details (disease duration, medication use, and smoking [past and present]) is filled out during screening.

### Qualitative Evaluation

Consecutive to the 12th session, participants are invited to a focus group to provide feedback and share their personal experiences with the program. The participants’ partners are also invited to the focus group. One week before the focus group, the coach instructs the participants to bring an object that symbolizes their experiences with the SALUD and to think about what they liked or disliked about the program. The focus groups will be video recorded and used for qualitative effect analysis of the program. In the video recording, all participants (and partners) are asked to provide informed consent to record the session and to use the video recording for qualitative analysis. The following sample question will be used:

- If you were the program director of SALUD, what would you change? Why?
- Would you recommend SALUD to other people? Why? Why not?
- What did SALUD mean to you? Which parts were valuable/useful for you?
- Which elements of SALUD were not valuable/useful for you?
- Did you change things in your everyday life because of SALUD (eg, daily life, interactions with others, and the way you think)? Why?

The video recording will be transcribed ad verbatim and pseudonymized. Two researchers of the research team analyze the transcripts independently. First, the transcripts are read and reread to engage with the data. Then, the transcripts are open coded on a descriptive and interpretative level. More specifically, transcripts will be coded on active components of the intervention, the mechanism of action, health-promoting resources, and the influence on everyday life. Subsequently, the codes are clustered in preliminary themes. Then, the 2 researchers compare and discuss the preliminary themes until consensus is reached. The overarching themes are the result of various discussions between the researchers. The themes will be displayed with pertinent participant quotes and detailed interpretative commentary. The Dutch quotes will be translated into English by a professional translator.

### Sample Size Calculation

Sample size calculation is based on the known mean value and SD of the SPFLS (food literacy measure) for the Dutch general population [36]. There are no RCTs with lifestyle interventions in the scientific literature that assessed food literacy (measured with SPFLS or another measure for food literacy) as a primary outcome. However, as RCTs evaluating lifestyle interventions that targeted psychosocial outcomes such as self-efficacy have demonstrated effect size of between 1.1 and 1.7 [48,49], it seemed reasonable to expect an effect size of 0.9. With use of the Cohen *d* calculation for effect size, an anticipated mean of 4.20 SPFLS score in the intervention group, and an anticipated mean of 3.83 (SD 0.41) in the control group is expected at T1 (based on references values [36]). With a standard power of 80% and a significance level of 5%, a total of 19 participants per group (38 participants in total) are needed. Adding a 30% dropout rate, the ideal sample size was set at 28 participants per group (56 in total).

### Statistical Analyses

Statistical analyses are carried out using IBM SPSS Statistics for Windows, version 28 (released in 2021; IBM Corp). Two-sided *P* values <.05 are regarded as statistically significant. Analyses are performed with the intention-to-treat principle, meaning that all patients were analyzed in their original allocation group regardless of the extent to which they followed the intervention. Dropouts are counted, and reasons for dropping out are recorded. If missing is completely at random, all available data at T0 (baseline), T1 (12 weeks), and T2 (24 weeks) are used to conduct the analyses. If missing is at random, multiple imputation techniques are used to impute the missing data.

Scores are calculated for multiple-item instruments (ie, DGSES, WHOQOL-BREF, SoC-13, DHD-15 index, and SPFLS). Randomization will be checked, and variables will be normalized if needed. Descriptive statistics are performed to tabulate mean (or median) values of all study characteristics and baseline values of the independent variables. Chi-square tests (for categorical variables) and 2-tailed *t* tests (for continuous variables) are used to compare the descriptive statistics between study groups and to identify potential covariates. Data are tabulated as mean (SD) or median (IQR) if skewed. Categorical data are presented as n (%) where appropriate.

According to a strict data analysis protocol, primary and secondary outcomes are tested with mixed models. Secondary outcomes are also tested if the primary outcome is not statistically significant. The analyses scripts will be written by the researchers; the analysis of the primary outcome will be run by an independent researcher who is blind to the intervention code. Assumptions for mixed models are checked. The mixed models will test the difference compared with baseline in the scores and values (ie, dependent variables) between intervention and control (ie, independent variable) at T1 and T2. Fixed effects are the treatment condition, time, and the covariates; random effects are the repeated measures per participant at T0, T1, and T2. Multiple models are tested; a crude model, and additional models that are adjusted for confounders commonly adjusted for in literature (eg, sex, age, education, and BMI). Variables that statistically significantly differed at baseline are also included in a model as covariates. The final results are presented in a table displaying the estimated marginal means, 95% CIs, and *P* values for the treatment effect for all primary and secondary outcomes.

### Incentive

All participants receive a financial compensation of €150 (US $159.5) after completion of the study (ie, after finishing the last measurements at T2). The financial compensation is calculated based on the time commitment and invasiveness of the measurements.

### Data Management

The handling of personal data complies with the European Union General Data Protection Regulation, the Dutch Act on Implementation of the General Data Protection Regulation (in Dutch: Uitvoeringswet Algemene Verordening Gegevensbescherming), and the Findability, Accessibility, Interoperability, and Reusability principles. Participants are assigned a unique participant identifier number (ie, a random 4 digits number) that does not change during the study to ensure that data cannot be tracked back to the participants. This number is linked with the name, address, and date of birth of the participant. The identifier number is used on raw and processed data files. The file that contains the link between the participant’s information and the participant’s identifier number is stored on a password-secured location during the study. Participants indicate in the informed consent form whether their data may be used for other studies. If a participant indicates that they do not allow further use of their data, their data will be removed from the file that can be requested for future research. The file with the link between the participant identifier number and the personal data of the participant will be destroyed after finalizing the data files for future research.

## Results

The project of which this study is part of received funding from the Edema-Steernberg Foundation in November 2016. Recruitment started on October 1, 2021. The intervention participants followed the SALUD program between January and August, 2022. The acquisition of the data was completed on August 1, 2022. The data analysis and publications are expected at the end of 2023.

## Discussion

This study will be one of the first salutogenic interventions for T2DM. Both the quantitative and the qualitative effect analyses are expected to provide valuable insights for further optimalization of interventions in people with T2DM.

### Strengths and Limitations

A first strength of this study includes the RCT design, especially because previous Dutch “diabetes reversal” interventions have been evaluated via an observational pre- and posttest design [4,50]. Furthermore, there is also a shortage of evaluating salutogenic programs via RCTs [51]. Although RCTs are considered the gold standard for intervention evaluation, the RCT design has also some limitations for this particular study. The SALUD program is difficult to fit the experimental framework of an RCT, as it is a multicomponent intervention that cannot be strictly standardized [52-54]. Furthermore, RCTs are limited when the implementation context is a determinant of the result [52-54]. In the SALUD study, the success of the program will be dependent on the extent to which the program integrates with the everyday lives of the participants. Nevertheless, RCTs can be helpful for evaluating multicomponent interventions if close attention is paid to contextual factors via qualitative analysis [54]. Hence, a second strength is the proposed qualitative evaluation. This will increases the value of this RCT as it may help to understand the “black box” of the SALUD program (eg, the active components of the SALUD intervention, participant satisfaction, and points of improvement) [54]. In addition, the qualitative evaluation together with the quantitative evaluation may gather new insights regarding the theory of salutogenesis [51,54]. A third strength is that participants can follow the program on the web in the comfort of their own home: it saves the participants’ time and travel efforts. Considering the recent COVID-19 pandemic, it is also a safe manner of group interactions, and the group sessions can continue during a possible lockdown. It also provides valuable information on how to perform a group-based coaching program in a web-based setting. A third strength is the open and flexible nature of the SALUD program, which provides the lifestyle coach opportunities to tailor the program to situational factors and individual needs. A final strength is the inclusion of both physical and psychosocial health measurements that enable assessment of the program’s effect on different dimensions of health. However, because of limited budget, the study does not include HbA_1c_ and blood measurements to assess T2DM management.

A limitation is the limited follow-up period. Ideally, measurements would be repeated over 1, 5, or even more years to determine whether the program led to sustainable change. However, the current Dutch university and funding structures make it difficult to execute such long-term research projects (eg, temporary research contracts). Challenges the study may face are the recruitment; owing to the COVID-19 pandemic, health care professionals are overflooded with work and are short staffed. Especially in the case of a new infection peak, health care professionals may be unable to assist in the recruitment of participants. Furthermore, the intervention might be specifically suited for more motivated people because of the time investment and because self-identity and self-reflection require a proactive attitude. This might cause dropouts in the intervention group.

### Conclusions

The proposed study will provide useful information on the effects of a salutogenic program on healthy eating and well-being in people with T2DM in a daily life setting. The SALUD program—if effective—may serve as a concrete, web-based tool to empower people with T2DM and guide them toward a healthier dietary pattern for the long run. In addition, the study may provide valuable information on what salutogenic interventions entail as well as new insights regarding the theory of salutogenesis. The combination of quantitative and qualitative measures allows a comprehensive evaluation of the intervention’s effects.

## Appendix

Multimedia Appendix 1 Peer review report and author comments from the Edema-Steernberg Foundation.

Multimedia Appendix 2 Peer review report and author comments from the Edema-Steernberg Foundation.

## Abbreviations

AHEAD: Action for Health in Diabetes
DGSES: Dutch General Self-Efficacy Scale
DHD-15 index: Dutch Healthy Diet Index 2015
DIRECT: Diabetes Remission Clinical Trial
GP: general practitioner
GRR: generalized resistance resource
HbA_1c_: hemoglobin A_1c_
QoL: quality of life
RCT: randomized controlled trial
SALUD: Salutogenic Intervention for Type 2 Diabetes
SoC: sense of coherence
SoC-13: 13 items of the orientation to life questionnaire
SPFLS: self-perceived food literacy scale
SRR: specific resistance resource
T2DM: type 2 diabetes mellitus
WHOQOL-100: World Health Organization Quality of Life-100
WHR: waist-hip ratio

## Data Availability

All pseudonymized data will be stored for 10 years on a password-protected server of Wageningen University. If other researchers wish to use the data, they have to send a request to the principal investigator. If the request is approved, the principal investigator will grant a copy of the file.
