# Supplementary material for: Effects of a Salutogenic Healthy Eating Program in Type 2 Diabetes (the SALUD Study): Protocol for a Randomized Controlled Trial
Source: JMIR Res Protoc. 2023 Mar 21;12:e40490. doi: 10.2196/40490 (PMC10131793; doi:10.2196/40490)
Supplement: Multimedia Appendix 2 [file resprot_v12i1e40490_app2.pdf]

Rebuttal to Reviewers

Funding organisation: Edema-Steernberg Foundation

Project	From diet to everyday-social life: a salutogenic approach towards enabling healthful eating among diabetes type 2 patients (Acronym: D2SAL)
Authors	Laura Bouwman, Sabita Soedamah-Muthu, Maria Koelen, Marianne Geleijnse

---

Pre-proposal fits in the scope of Edema Steernberg's call for proposals. Why are people [in this case Type 2 Diabetes patients (T2DM)] not following dietary recommendations in daily life. It is hypothesized that recommendations have insufficient eye for the everyday life context of people with T2DM. Focus is to identify and apply resources to cope with stressors challenging healthful eating.

Comment 1. The interplay of resources (self-efficacy, cooking skills, flexibility) and the cumulative learning effect of life-experiences in both childhood and adulthood is interesting. But next to personal attributes, other supportive resources (social, environmental assets) needs to be addressed as well. This way the research would fit better in the concept "why do people eat, what they eat".

Authors reply 1. We agree with the reviewers that besides personal, social and environment assets are crucial to enable dietary change. Hence, we added the validated method of Assets Mapping to indicate resources within the social and physical environment.
The Mediterranean diet that is used in the project as the 'exemplary healthy diet' features the socially embedded nature of eating, described by UNESCO as '*involving a set of skills, knowledge, rituals, symbols and traditions concerning crops, harvesting, fishing, animal husbandry, conservation, processing, cooking, and particularly the sharing and consumption of food. Eating together is the foundation of the cultural identity and continuity of communities throughout the Mediterranean basin. It is a moment of social exchange and communication, an affirmation and renewal of family, group or community identity'.* This represents everyday life values on food production, processing, sharing, communication, cultural tradition and – knowledge.

Comment 2. The selection of the Mediterranean needs to be explained, why not a diet according to the new Dutch dietary guidelines?

Authors reply 2. We would like to clarify that the Dutch Dietary guidelines are central in this project and have changed our wording in the proposal. The recent guidelines recommend a more plant based diet and less animal based diet. Several dietary patterns, including the Mediterranean diet, were found to be suitable to recommend as healthy diet. The Mediterranean diet reflects not only a more plant based diet, but is also described as a way of living including social and environmental aspects (see author reply to 1]). The unique collaboration between nutritional and social sciences within our project is in line with the UNESCO definition.
Of all dietary patterns, Mediterranean diet is the only diet with sound scientific evidence from large randomised trials to investigate the effect of the Med diet on long-term diabetes and cardiovascular diseases. The landmark Predimed trial demonstrated that Med diet is effective in Mediterranean countries. Therefore, it will have great potential to translate this diet into the Dutch situation for reversal of diabetes. We are collaborating with the Predimed investigators from Spain on this project. The challenge of our study will be to introduce and maintain this diet in the Dutch situation. Nowadays, products and recipes from all over the world are offered in the Netherlands, making it easier to supply this diet.

Comment 3. Operationalization of the different variables in quantitative terms will be a challenge. Moreover design, compliance and statistical power of the 2x2 trial needs careful attention.

Authors reply 3. The design, compliance and statistical power are now described in the proposal. We decided to investigate the effectiveness of the social behavioural tools with Med diet in a randomized parallel design as the most effective design to study reversal of diabetes. The trial will be imbedded in the infrastructure of GP practices in Ede. All measurements will be

performed within the existing infrastructure during scheduled visits of recently diagnosed type 2 diabetes patients.

Comment 4. Not really clear is how this validated toolbox will look like. It should not be restricted to a number of “tips and tricks”.

Authors reply 4. The toolbox will include validated instruments targeting active engagement in healthful eating that is considered the starting point for changing dietary patterns in the Salutogenic Model of Health. The tools will be specified to the everyday-life of T2DM within the project. A more detailed description is enclosed in the proposal.

Comment 5. Better understanding of how the interplay is of resources and cumulative learning has first priority.

Authors reply 5. The interplay of resources and cumulative learning is now described in the introduction of the grant proposal. We have included a more detailed description and a figure that visualizes the interplay.

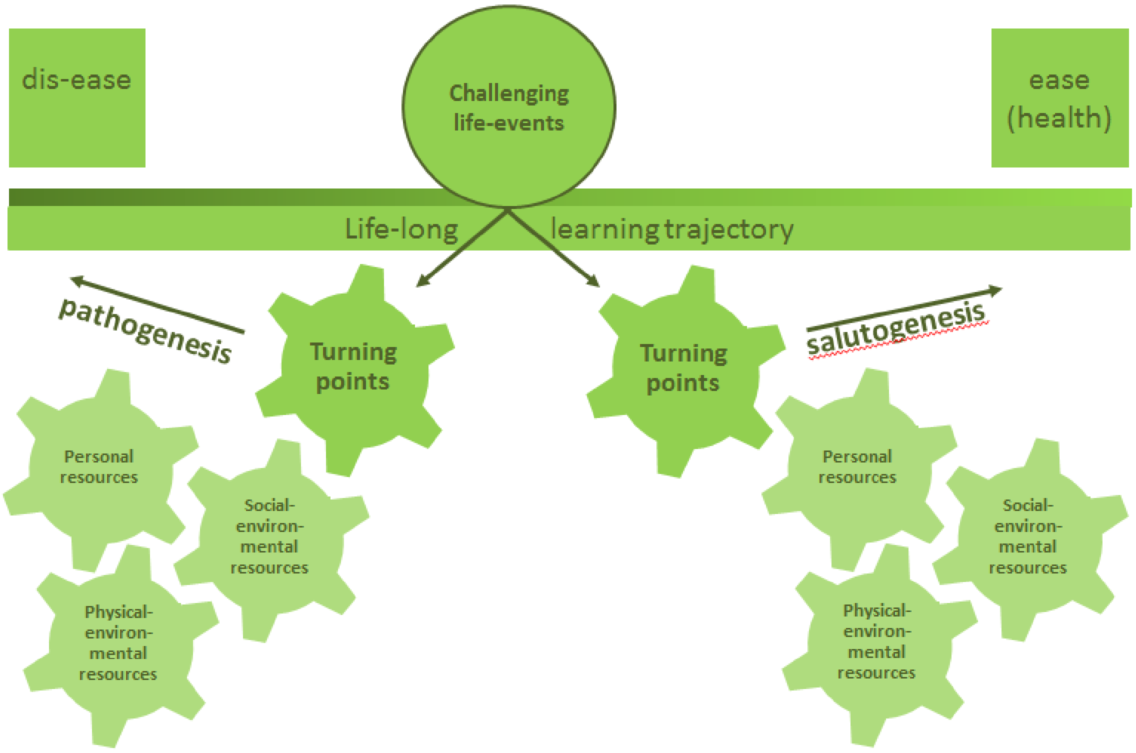

Comment 6. The role of all organisations involved should be made clear in the full proposal

Authors reply 6. We have included a more detailed description of the role of the national and international collaborators in the project.
