# Supplementary material for: Effects of a Salutogenic Healthy Eating Program in Type 2 Diabetes (the SALUD Study): Protocol for a Randomized Controlled Trial
Source: JMIR Res Protoc. 2023 Mar 21;12:e40490. doi: 10.2196/40490 (PMC10131793; doi:10.2196/40490)
Supplement: Multimedia Appendix 1 [file resprot_v12i1e40490_app1.pdf]

Rebuttal to Reviewers : research proposal 'From diet to everyday-social life'

Laura Bouwman, Sabita Soedamah-Muthu, Marianne Geleijnse and Maria Koelen.

We would like to thank the reviewers for the detailed and positive evaluation of our proposal and providing constructive comments to improve our work. Please find our responses below.

Reviewer 1.

R1-C1. *What is the aim of the systematic review; how is this review related to the SMH and the everyday-life perspective?*

The review aims to identify the available best practices ('what') and processes ('how to') with regard to supporting adults diagnosed with a diet-related chronic illness in accomplishing dietary change.

General criteria:

1) Aim to reverse T2DM through dietary and behavioural intervention;
2) Age > 18;

Criteria specific to the SMH and everyday-life perspective:

3) Target self-management capacity;
4) Address practical and social organisation of healthful eating in everyday-life;
5) Apply a comprehensive set of physiological (including nutritional) and psycho-social outcome measures.

R1-C2. *How will you deal with the data from the narrative inquiry when participants have different life events and responses to it.*

The analysis is focused on how participants develop self-management capacity when faced with life-events, how such events develop to turning points in dietary patterns and the resources they identify and apply. The results will provide insight in the connection between certain situations, turning points and resources. This informs the mechanisms that should be set in motion by the learning trajectory provided in the RCT.

R1-C3. *How can stage 1 and stage 2 data be integrated, given the differences in study design and population characteristics?*

The selection criteria of study populations (T2DM, age range) are the same for stage 1 and 2.

We would like to extract from stage 1 which intervention designs are effective and which behavioural aspects are most crucial to address in stage 2. In stage 2, the learning processes underlying the behavioural aspects are derived through in-depth interviews. Both outcomes will be integrated to design the intervention for stage 3.

R1-C4. *Is the power of the study sufficient and what is the protocol of the '+everyday-life intervention'*

The sample size of 25 mentioned by the reviewer is per group. We will include a total of 50 patients. More details on the power of the study are given in R2-C2.

<u>Protocol</u>: The protocol will be developed during the project when we gained insight in the practices (what to do) and processes (how to do it) relevant to T2DM.

Reviewer 2.

R2-C1. *Will current attention to 'what works' and patients' life situation in regular counselling be considered?*

Most counselling nowadays is targeted on patients' life situation. However, to our knowledge it still does not fully address the practical and social organisation of change within the everyday-context that involves more than 'diet and health'. Also, current counselling does target patient self-management, however merely overlooks the need for the development of everyday-life agency and sensitivity to the ever changing context. The latter requires active learning rather than the current provision of (tailored) information.

R2-C2. *Inclusion criteria leave little room for improvement; recalculation of power*

Staying closer to real life patients we decided not to restrict HbA1c levels to <53 mmol/mol at baseline, but include patients who are stable on medication as randomisation will balance this out between intervention groups. Including type 2 diabetes patients with varying HbA1c levels at baseline will give more room for improvement in HbA1c and glucose levels during the course of the study.

<u>Power</u>: In behavioural intervention studies in diabetic patients, sample sizes ranged from 20-40. These numbers were sufficiently large to demonstrate an effect on glucose/HbA1c levels with a power of 80% and two-sided α of 0.05 (Noh 2010; Smith 1997). Our study effects will be based on a combination of

diet and new behavioural strategies, which have not been used previously. With the 6 months follow-up period in our study, a larger effect can be expected compared to the usual 3 months follow-up period in other studies.
The effects of combining these interventions is stronger than studies which use either dietary or behavioural interventions. We do not only focus on glucose control, but also on improvements in dietary quality (Med Diet Score) which have larger effects, are easier to achieve and for which we have ample power. With a large General Practice source population of >1,000 type 2 diabetes patients in the Ede region, we are confident we will find a sample size of 50.

Reviewer 3.
R3-C1. *Consideration of the practicalities of recruitment for the clinical asp*ects
Patients with type 2 diabetes will be recruited using the existing infrastructure of the GP practices in Ede, the Netherlands. These practices include a source population of >1,000 type 2 diabetes patients in the Ede region which is more than sufficient for our required sample size. All type 2 diabetes patients visit the nurse practitioner at 3-monthly intervals for their usual care. During these visits information on our project will be handed out to type 2 diabetes patients meeting our inclusion criteria. All nurse practitioners will be trained centrally to do this in a standardized manner. If patients would like to participate in the study, they can contact us and informed consent forms will be signed. For the next visit to the nurse practitioner, study participants will be asked to fast overnight for blood collections. We already made arrangements with the central laboratory in the local hospital where usually bloods are taken from GP practices in the region. A standard blood form for research purposes can be used to get our samples processed and fasting glucose and glycated haemoglobin will be measured in the hospital's central laboratory with standardized methods. Questionnaires, dietary instructions and the every-day life perspective advice will be provided by the PhD student and dietician at the GP practices and budget for travel and room hire has been reserved. An equal number of contact moments will be provided to both the intervention and control group.

R3-C2. *Epistemological considerations required for mixed methods in which pathogenic and salutogenic perspectives are combined*
The SMH in itself is a 'mixed' framework. It presents health as a continuum and studies focus on the risks leading to a movement towards 'dis-ease' (pathogenic) as well as resources supporting movements towards 'ease' (salutogenesis). Hence, data derived from methods rooted in the scientific, interpretative and critical paradigm all fit under this framework.
The ontological and epistemological positions that underlie the methods in the study will be considered extensively. We consider the use of Integral Ecology's AQAL framework to position the different types of data in the study that stem from the scientific, interpretative and critical paradigms. AQAL has been previously applied in biomedical/social-behavioural studies towards health-related phenomena to position and work towards data integration (Esbjorn-Hargens and Zimmerman, 2009).

R3-C3. *Does a PhD trajectory accommodate acquisition of required interdisciplinary competences?*
Recruitment criteria will include an interdisciplinary background, such as an MSc in Nutrition with a minor/thesis in the social sciences or vice versa (which is quite common at Wageningen University). The PhD student will be offered courses at the Wageningen Graduate schools VLAG (food & nutrition) and WASS (social sciences) to upgrade knowledge and skills for the present project.

References
Esbjorn-Hargens, S., & Zimmerman, M.E. (2009). *Integral Ecology: uniting multiple perspectives on the natural world*. Boston: Integral Books.

Noh J-H, Cho Y-J, Nam H-W, Kim J-H, Kim D-J, Yoo H-S, Kwon Y-W, Woo M-H, Cho J-W, Hong M-H, Yoo J-H, Gu M-J, Kim S-A, An K-E, Jang S-M, Kim E-K, and Yoo H-J (2010). Web-Based Comprehensive Information System for Self-Management of Diabetes Mellitus. Diabetes Technology & Therapeutics. 12: 333-337.

Smith DE, Heckemeyer CM, Kratt PE, Mason DA. Motivational interviewing to improve adherence to a behavioral weight-control program for older obese women with NIDDM (1997) Diabetes Care: 20:52.
